# TEMPO-Oxidized Cellulose Nanofiber-Alginate Hydrogel as a Bioink for Human Meniscus Tissue Engineering

**DOI:** 10.3389/fbioe.2021.766399

**Published:** 2021-11-05

**Authors:** Xiaoyi Lan, Zhiyao Ma, Alexander R. A. Szojka, Melanie Kunze, Aillette Mulet-Sierra, Margaret J. Vyhlidal, Yaman Boluk, Adetola B. Adesida

**Affiliations:** ^1^ Department of Civil and Environmental Engineering, University of Alberta, Edmonton, AB, Canada; ^2^ Divisions of Orthopaedic Surgery and Surgical Research, Laboratory of Stem Cell Biology and Orthopaedic Tissue Engineering, Department of Surgery, University of Alberta, Edmonton, AB, Canada

**Keywords:** tissue engineering, meniscus, 3D bioprinting, cellulose nanofiber, hypoxia

## Abstract

**Objective:** The avascular inner regions of the knee menisci cannot self-heal. As a prospective treatment, functional replacements can be generated by cell-based 3D bioprinting with an appropriate cell source and biomaterial. To that end, human meniscus fibrochondrocytes (hMFC) from surgical castoffs of partial meniscectomies as well as cellulose nanofiber-alginate based hydrogels have emerged as a promising cell source and biomaterial combination. The objectives of the study were to first find the optimal formulations of TEMPO (2,2,6,6-tetramethylpiperidine-1-oxyl)-oxidized cellulose nanofiber/alginate (TCNF/ALG) precursors for bioprinting, and then to use them to investigate redifferentiation and synthesis of functional inner meniscus-like extracellular matrix (ECM) components by expanded hMFCs.

**Methods:** The rheological properties including shear viscosity, thixotropic behavior recovery, and loss tangent of selected TCNF/ALG precursors were measured to find the optimum formulations for 3D bioprinting. hMFCs were mixed with TCNF/ALG precursors with suitable formulations and 3D bioprinted into cylindrical disc constructs and crosslinked with CaCl_2_ after printing. The bioprinted constructs then underwent 6 weeks of *in vitro* chondrogenesis in hypoxia prior to analysis with biomechanical, biochemical, molecular, and histological assays. hMFCs mixed with a collagen I gel were used as a control.

**Results:** The TCNF/ALG and collagen-based constructs had similar compression moduli. The expression of *COL2A1* was significantly higher in TCNF/ALG. The TCNF/ALG constructs showed more of an inner meniscus-like phenotype while the collagen I-based construct was consistent with a more outer meniscus-like phenotype. The expression of *COL10A1* and *MMP13* were lower in the TCNF/ALG constructs. In addition, the immunofluorescence of human type I and II collagens were evident in the TCNF/ALG, while the bovine type I collagen constructs lacked type II collagen deposition but did contain newly synthesized human type I collagen.

## Introduction

The menisci are a pair of C-shaped fibrocartilages that withstand compressive and tensile forces (Fithian et al., 1990; Leslie et al., 2000; Liang et al., 2018). They are essential for mechanical load distribution and transmission, lubrication, and stability of the knee joint (Makris et al., 2011; Liang et al., 2018). The biomechanical properties are attributed to the presence of functional extracellular matrix (ECM) (Fithian et al., 1990; Leslie et al., 2000; Adesida et al., 2007; Liang et al., 2018). The menisci exhibit regional and zonal variations in their ECM and cellular compositions, reparative capacities, and vascularity with age (Arnoczky and Warren, 1982; Nakata et al., 2001; Chevrier et al., 2009; Sanchez-Adams and Athanasiou, 2009). Type I collagen (collagen I) is found throughout the entire meniscus; type II collagen (collagen II) and aggrecan are usually only found in the inner regions of the meniscus (King, 1936; Nakata et al., 2001; Szojka et al., 2021). The outer region of the meniscus contains collagen I fibre bundles that aligned in the circumferential direction, exhibiting a fibrous connective tissue (ligament and tendon-like) phenotype and a peripheral blood supply (1/3 or less in adults) that provides some healing capacity (King, 1936; Nakata et al., 2001; Szojka et al., 2021). The cell population in the outer meniscus is predominantly fibroblast-like. In contrast, the middle and inner regions (2/3 or more) of the meniscus have a fibrocartilage phenotype that is avascular and non-healing (King, 1936; Nakata et al., 2001; Szojka et al., 2021). The cell population for the inner region of the meniscus are human meniscus fibrochondrocytes (hMFC), which are a mix of fibroblast and chondrocyte-like cells (King, 1936; Nakata et al., 2001; Szojka et al., 2021).

Due to the limited healing capacities of meniscus, patients with meniscus defects or injuries often undergo partial or total meniscectomy, which is known to cause biomechanical changes to joint function with a risk for early knee osteoarthritis development (Fairbank, 1948; Cox et al., 1975; Paradowski et al., 2016). Cell-based meniscus tissue engineering is a promising technology to circumvent this challenge by creating meniscus tissue replacements (Ibarra et al., 2000). By using 3D bioprinting with a “bioink” composed of a biomaterial and cells, can create patient-specific tissues for reconstructive surgery (Murphy and Atala, 2014; Di Bella et al., 2015; Daly et al., 2017). In fact, 3D bioprinting can spatially control the placement of cells, biomaterial, and growth factors in a construct. Therefore, it has the potential to accurately mimic the structure and morphology of tissues and organs (Murphy and Atala, 2014; Mandrycky et al., 2016). Natural hydrogel precursors are attractive for bioinks because of their favourable biocompatible properties and high-water content like human ECM (Markstedt et al., 2015; Skardal et al., 2015; Ávila et al., 2016). To be suitable for printing, the rheological behaviour of the bioink is critical (Paxton et al., 2017; Cooke and Rosenzweig, 2021). The bioink must have shear thinning properties, which allows extrusion through small orifices with a decrease in shear to maintain cell viability. The bioink also needs a high zero-shear viscosity to retain its shape during and after printing (Paxton et al., 2017; Cooke and Rosenzweig, 2021). Beyond rheological properties, the bioink needs to be crosslinkable to retain 3D structures and provide appropriate mechanical properties to the bioprinted constructs (Markstedt et al., 2015; Paxton et al., 2017). Alginate has been proven as a promising crosslinkable material for 3D bioprinting (Markstedt et al., 2015; Müller et al., 2017; Nguyen et al., 2017; Gatenholm et al., 2018; Göhl et al., 2018; Hiller et al., 2018; Wu et al., 2018; Yang et al., 2018; Heggset et al., 2019; Jessop et al., 2019; Ojansivu et al., 2019; Erkoc et al., 2020; Schwarz et al., 2020). It can form a stable hydrogel in the presence of divalent cations such as Ca^2+^ and Ba^2+^ due to the ionic interaction between the cation and the carboxyl functional group by forming “egg-box”-calcium linked junctions (Li et al., 2007a). Alginate is usually mixed with other biomaterials to achieve higher printing resolution (Markstedt et al., 2015; Müller et al., 2017; Nguyen et al., 2017; Heggset et al., 2019; Jessop et al., 2019; Erkoc et al., 2020).

Cellulose, the most abundant renewable biopolymer found in nature, is a linear polysaccharide composed of *β* (1→4) linked D-glucose units (Dufresne, 2013; Abitbol et al., 2016; Abdul Rashid et al., 2018). The term *nanocellulose* refers to processed cellulose extract with one dimension in the nanometer range (Dufresne, 2013; Abitbol et al., 2016; Gatenholm et al., 2018). Depending on the sources and the preparation method, nanocellulose materials can be categorized into three main groups: bacteria cellulose (BC), cellulose nanofiber (CNF), and cellulose nanocrystal (CNC) (Wang et al., 2020b). The most common nanocellulose used in 3D bioprinting applications is CNF (Wang et al., 2020b). CNFs exhibit shear-thinning behaviour and a high zero shear viscosity, making them useful as a viscous contributor for the bioink. CNFs can be extracted from raw materials by a combination of chemical (e.g., acid hydrolysis, enzymatic reaction, TEMPO oxidation) and mechanical treatments (e.g., high-pressure homogenization and grinding) (Wang et al., 2020b). TEMPO-oxidized CNFs (TCNFs) exhibit a high concentration of carboxyl groups and the new TEMPO-induced carboxyl groups in TCNFs are able to support alginate in the construction of crosslinked network, which enhances the scaffold mechanical strength, porosity, water absorption, and structural integrity (Lin et al., 2012).

**FIGURE 1:**
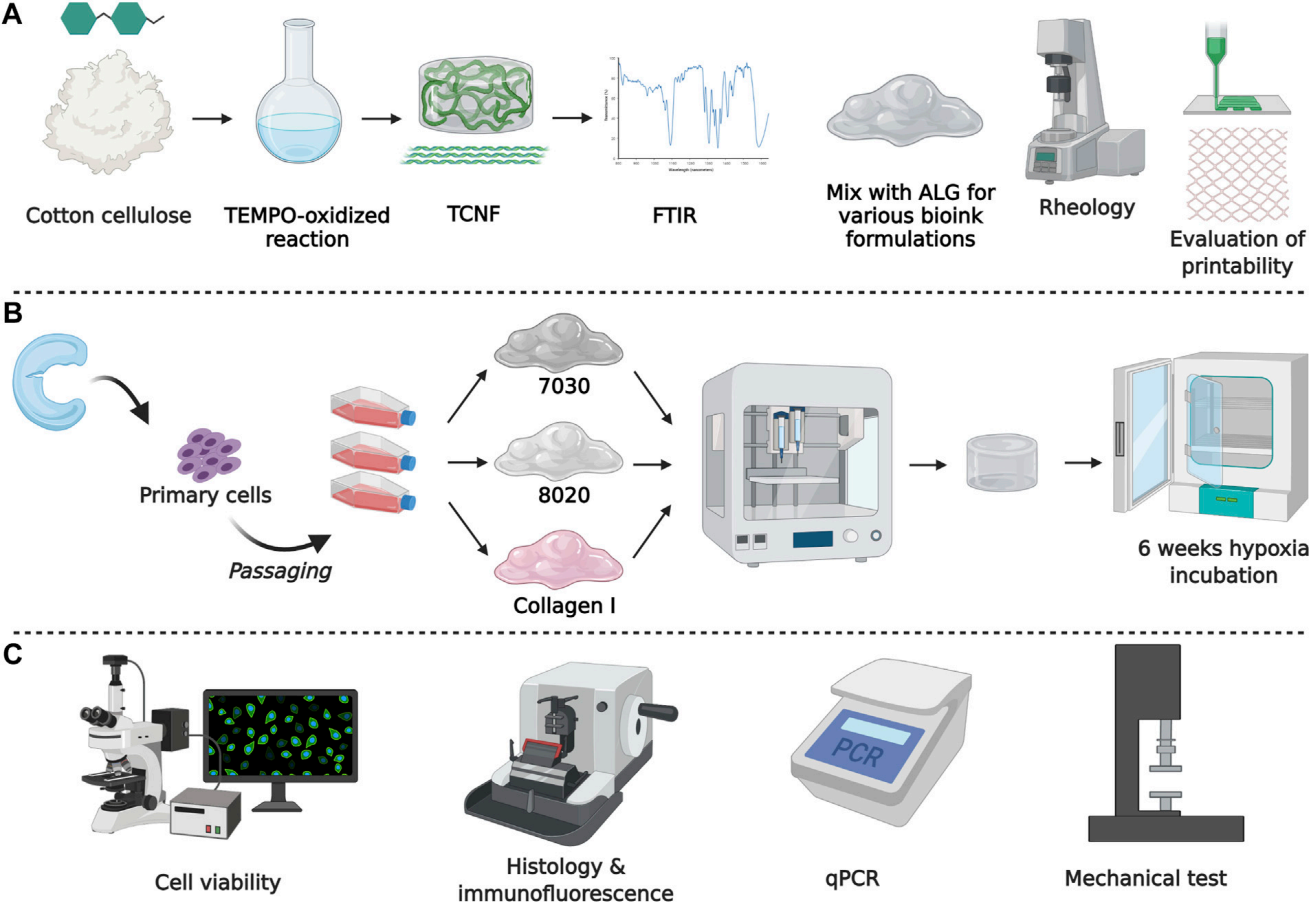
Schematic diagram of the experimental design. **(A)** Biomaterial formation and characterization, **(B)** engineered tissue formation, and **(C)** evaluation of engineered tissues.

This study incorporates hMFCs with TEMPO-oxidized CNF and alginate (TCNF/ALG) precursors to create tissue-engineered meniscus constructs. First, we evaluated the printability and the rheology properties of various formulations of TCNF/ALG precursors as potential bioinks. Then, we evaluated the biological functionality of 3D bioprinted meniscus-like tissue constructs using the optimal formulations, followed by 6 weeks of *in vitro* chondrogenic culture in low oxygen tension (i.e., hypoxia of 3% O_2_). Collagen I, the major component of the meniscus fibrocartilage’s ECM, served as a reference biomaterial. Hypoxia was used since it has been proven as a stimulus for the maturation of meniscus fibrochondrocytes (Herrera Millar et al., 2021). Our previous research has found that hypoxia and TGF-β3 synergistically mediated the inner meniscus-like tissue matrix formation (Szojka et al., 2019). The schematic diagram of the experimental design is shown in Figure 1.

## Materials and Methods

### TCNF Synthesis and Preparation of TCNF/ALG Precursors

Native cellulose (10 g, Whatman No. 1 filter paper) was cut into small pieces and immersed in deionized water (DI) water to make 1 L of 1% w/w cellulose pulp slurry. In addition, 0.189 g of TEMPO (Sigma Aldrich, Canada), 1.178 g NaBr (Sigma Aldrich, Canada), and 50 ml of 1.8 M NaClO (Sigma Aldrich, Canada) were added into the cellulose slurry under continuous stirring until dissolved. The pH of the reactants in the flask was maintained at pH = 10.5 by adding 0.5 M NaOH until the pH of the reactant were stable at 10 (Shinoda et al., 2012). The TEMPO-oxidized cellulose was then washed with DI water for 15 times until the conductivity for supernatant was constant (∼12.5 µs/cm). The TEMPO-oxidized cellulose nanofiber (TCNF) was prepared by blending the TEMPO-oxidized cellulose for 7 min. The carboxylate content for the TCNF was 0.84 mmol/g. The TCNF was centrifuged and adjusted to 3.5% w/v solid content. Freeze-dried cellulose pulp slurry (pure cellulose) and TCNF were characterized by Fourier Transform Infrared spectroscopy (FTIR).

Sodium alginate (ALG, Alfa Aesar, J61887, United States) was prepared into a 3.5% w/v solution. The concentrated TCNF and the ALG solution were sterilized by autoclave. The TCNF and ALG are mixed into various printing formulations (Table 1) with a final solid content of 3.5% w/v.

**TABLE 1:** TCNF/ALG precursor formulations.

Precursors	Composition	Formulation (% w/v)	Solid content (% w/v)
0100	100% of ALG	ALG: 3.5	3.5
2080	20% TCNF	TCNF: 0.7	3.5
	80% ALG	ALG: 2.8	
5050	50% TCNF	TCNF: 1.75	3.5
	50% ALG	ALG: 1.75	
6040	60% TCNF	TCNF: 2.1	3.5
	40% ALG	ALG: 1.4	
7030	70% TCNF	TCNF: 2.45	3.5
	30% ALG	ALG: 1.05	
8020	80% TCNF	TCNF: 2.8	3.5
	20% ALG	ALG: 0.7	
9010	90% TCNF	TCNF: 3.15	3.5
	10% ALG	ALG: 0.35	

### Rheological Characterization of TCNF/ALG Precursors as Potential Bioink Materials

The rheological properties of all the TCNF/ALG precursors were characterized using a rotatory rheometer (AR-G2, TA Instrument, United States) with a 25 mm parallel-plate geometry. The steady-state flow sweep was characterized under the shear rate from 0.001 to 1,000 s^−1^ at room temperature. The oscillatory frequency sweep was measured under 1% strain (within the linear viscoelastic region) and 1–100 rad/s frequency. The thixotropy tests were done by first fixing the shear rate at 1 s^−1^ for 100 s, followed by a sudden increase in the shear rate to 1,000 s^−1^, and then a sudden drop in the shear rate to the initial state (1 s^−1^). The rheology data was analyzed using TRIOS software (TA Instruments, United States).

### Assessment of Printing Fidelity Before and After Crosslinking

To assess the printability of the TCNF/ALG precursors, 20 mm × 20 mm × 3 mm blocks with a 30% infill were bioprinted using an extrusion-based bioprinter (INKREDIBLE+, CELLINK, Sweden). The geometry and printing parameters of the printed constructs were predefined in commercial design software (Slic3r, United States). The needle inner diameter was 0.413 cm (22G). The printing speed was 10 mm/s with each layer height of 0.5 mm (6 layers in total). After bioprinting, a 100 mM CaCl_2_ solution was added over the bioprinted TCNF/ALG constructs for 3 min followed by a phosphate buffered saline (PBS) rinse. After crosslinking, the precursors with the best printing fidelity were used for further biological functionality evaluation. A commercially available bovine collagen I gel with similar solid content to the TCNF/ALG precursors was used as a control (3.5% w/v, Advanced Biomatrix, United States). The collagen I gel was printed at room temperature and spontaneously polymerized at 37°C. The fidelity parameter (side length of the square blocks (L) before and after the crosslinking, filament diameter) were quantified using ImageJ (Fiji, United States). The contractility from the crosslinking process was calculated as:

$$\mathrm{contractility}=\left(L_{\mathrm{before crosslinking}}-L_{\mathrm{after crosslinking}}\right)/L_{\mathrm{before crosslinking}}$$

### Isolation of Human Meniscus Fibrochondrocytes

Meniscus specimens from three (3) male donors were collected from partial meniscectomy surgeries with the approval of the University of Alberta’s Health Research Ethics Board - Biomedical Panel (Study ID: Pro00018778). Human meniscus fibrochondrocytes (hMFCs) were isolated enzymatically using collagenase digestion as previously described (Liang et al., 2017). The primary hMFCs were plated at a density of 10^4^ cells/cm^2^ and expanded to passage 2 (P2) in monolayer culture in high glucose Dulbecco’s Modified Eagle Medium (DMEM, Sigma Aldrich, Canada) supplemented with 10 v/v (%) fetal bovine serum (FBS), 1 ng/ml of TGF-β1, and 5 ng/ml of FGF-2 at 37°C in normoxia (∼20% O_2_) conditions (Liang et al., 2017). Donor information is shown in Table 2. The cumulative population doublings of the hMFCs at the end of P2 was 7.0 ± 0.2 (mean ± standard deviation).

**TABLE 2:** Meniscus donor information.

Donor	Sex	Age	Medical history	Anatomical site	Cumulative population doubling
1	Male	30	ACL reconstruction	Right knee medial	7.0
2	Male	27	Healthy	Left knee medial	7.3
3	Male	16	ACL tear	Right knee lateral	6.8

### 3D Bioprinting of TCNF/ALG and Collagen Bioinks

hMFCs at P2 were resuspended in a standard serum-free chondrogenic medium containing 10 ng/ml TGF-β3 (Liang et al., 2017). The cell suspension was mixed with 7030, 8020 precursors (7030, 8020 bioinks), and collagen I gel (COL bioink) to a cell density of 10^7^ cells/mL.

To investigate the fibrocartilage formation of the bioprinted constructs after *in vitro* chondrogenic culture, hMFC-laden TCNF/ALG bioinks and COL bioink were bioprinted into cylindrical shapes (7 mm diameter, 3.5 mm height) with an infill rate of 70%. Then bioprinted constructs were crosslinked as before. At the same time, the hMFC-laden COL bioinks were kept in a 37°C incubator for 30 min and then placed in a serum-free chondrogenic medium containing 10 ng/ml TGF-β3 (2 ml/construct, twice per week) for 6 weeks under hypoxic conditions (3% O_2_).

### Viability in Bioprinted Constructs

hMFCs viability was assessed using Syto 13/Propidium iodide (PI) staining. Syto 13 (S7575, ThermoFisher, United States) is a green live-cell fluorescent nucleic acid stain, and PI (P3566, ThermoFisher) is a red dead-cell fluorescent nuclear and chromosome counterstain. The Syto 13 and PI concentrations in PBS were 6.25 and 15.0 μM, respectively.

Bioprinted constructs of TCNF/ALG (8020, 7030 bioinks) and COL bioinks after 1-day culture were incubated in Syto 13 and PI solution at room temperature for 30 min in the dark. The cell viabilities were viewed under a Nikon confocal laser scanning microscope (Leica TCS SP5). Fluorescence was quantified using Python.

### Structural Integrity and Microstructural Evaluation of Bioprinted Constructs

Scanning electron microscopey (SEM., Zeiss Sigma 300 VP-FESEM) was used to observe the TCNF fibre diameter and the ECM formation of the bioprinted constructs after 6 weeks of *in vitro* culture. Each construct was fixed in sodium cacodylate trihydrate buffer containing 2% v/v glutaraldehyde and 2.5% v/v paraformaldehyde overnight. The samples were then cut in half and washed twice with deionized water to wash away the fixation solution. The samples were further treated with osmium tetroxide and tannic acid before SEM observation. All the reagents were purchased from Electron Microscopy Sciences (Pennsylvania, United States).

### Histological and Immunofluorescent Evaluation of Matrix Formation

Bioprinted constructs for three experiment groups (8020, 7030, COL) from the same donor were fixed in 10% (v/v) neutral buffered formalin, dehydrated through a series of alcohol washes, and then embedded in paraffin. The embedded samples were cut into 5 μm sections cross-sectionally.

For histological assessments, the sections were deparaffinized by the xylene substitute, then rehydrated and stained with Safranin-O/Fast Green. Collagens I, II, and X as well as aggrecan were examined by immunofluorescence. In brief, sections were prepared as above. Collagens I and II were counterstained in one slide. Collagen X and aggrecan were stained separately in two slides. The primary antibodies for collagens I and II were rabbit anti-human collagen I (CL50111AP-1, Cedarlane, Canada) and mouse anti-human collagen II (II-II6B3, Develop-mental Studies Hybridoma Bank [DSHB], United States). The primary antibody for type X collagen (collagen X) was rabbit anti-human collagen X (rabbit polyclonal to collagen X, ab58632, Abcam, United States), and the primary antibody for aggrecan was a rabbit anti-human aggrecan (recombinant monoclonal to aggrecan, MA5-32695, ThermoFisher, United States).

After incubation with primary antibodies, the slides were incubated with secondary antibodies for 45 min. The secondary antibodies used for collagens I and II are goat anti-rabbit IgG Alexa Fluor 594 (ab150080, Abcam, United States) and goat anti-mouse IgG Alexa Fluor 488 (ab150117, Abcam, United States). The secondary antibodies used for collagen X and aggrecan were goat anti-rabbit IgG Alexa Fluor 594 (ab150080, Abcam, United States). In addition, all the slides were also stained with 4′,6-diamidino-2′- phenylindole (DAPI, Thermo Fisher Scientific) for 20 min at room temperature to examine the nuclei of hMFCs in each sample. A Nikon Eclipse Ti-S microscope coupled to a DS-U3/Fi2 Color CCD camera with 100x and 200x total magnification was used to capture the histological and immunofluorescent images.

### Gene Expression Analysis

Quantitative real-time polymerase chain reaction (qRT-PCR) was used to analyze expression of genes that are chondrogenic and fibrochondrogenic specific (*ACAN, COL1A2, COL2A1, SOX9*) and chondrocyte hypertrophy-related (*COL10A1, MMP13, ALPL, RUNX2*). The analysis was conducted after 6 weeks (42 days) of *in vitro* chondrogenic culture. Total RNA was extracted using TRIzol (Life Technologies, United States). The complementary DNA (cDNA) was synthesized from 100 ng of total RNA using GoScript, Reverse Transcriptase kit (Promega, United States) and 1 µg of oligo (dT) primer. Primer sequences for qPCR were designed using Primer Express 3.0.1 (Thermo Fisher Scientific). Transcript levels for the interested genes were normalized to the housekeeping genes: *β-actin*, *B2M*, and *YWHAZ* using the delta CT method (2^−*ΔCT*^). Primer sequences are shown in Table 3.

**TABLE 3:** Primer sequences for quantitative real-time polymerase chain reaction.

Gene	Forward primer (5′)	Reverse primer (3′)
*Β-actin*	AAG​CCA​CCC​CAC​TTC​TCT​CTA​A	AAT​GCT​ATC​ACC​TCC​CCT​GTG​T
*B2M*	TGC​TGT​CTC​CAT​GTT​TGA​TGT​ATC​T	TCT​CTG​CTC​CCC​ACC​TCT​AAG​T
*YWHAZ*	TCT​GTC​TTG​TCA​CCA​ACC​ATT​CTT	TCATGCGGCCTTTTTCCA
*ACAN*	AGG​GCG​AGT​GGA​ATG​ATG​TT	GGT​GGC​TGT​GCC​CTT​TTT​AC
*COL1A2*	GCT​ACC​CAA​CTT​GCC​TTC​ATG	GCA​GTG​GTA​GGT​GAT​GTT​CTG​AGA
*COL2A1*	CTG​CAA​AAT​AAA​ATC​TCG​GTG​TTC​T	GGG​CAT​TTG​ACT​CAC​ACC​AGT
*SOX9*	CTT​TGG​TTT​GTG​TTC​GTG​TTT​TG	AGA​GAA​AGA​AAA​AGG​GAA​AGG​TAA​GTT​T
*COL10A1*	GAA​GTT​ATA​ATT​TAC​ACT​GAG​GGT​TTC​AAA	GAG​GCA​CAG​CTT​AAA​AGT​TTT​AAA​CA
*RUNX2*	GGA​GTG​GAC​GAG​GCA​AGA​GTT​T	AGC​TTC​TGT​CTG​TGC​CTT​CTG​G
*VCAN*	TGC​TAA​AGG​CTG​CGA​ATG​G	AAA​AAG​GAA​TGC​AGC​AAA​GAA​GA
*MMP13*	AAA​AAG​GAA​TGC​AGC​AAA​GAA​GA	CGG​AGA​CTG​GTA​ATG​GCA​TCA
*ALPL*	GCT​GTA​AGG​ACA​TCG​CCT​ACC​A	CCT​GGC​TTT​CTC​GTC​ACT​CTC​A

### Biomechanical Characterization by Stress Relaxation Tests

The mechanical properties of bioprinted constructs were assessed using a stepwise stress relaxation test using a Biodynamic 5,210 system (TA Instruments, United States). For each experimental group, constructs from *n* = 3 donors were tested. The constructs were preconditioned by 15 cycles of sine wave dynamic compressive loading with an amplitude of 5% tissue height at a frequency of 1 Hz. The following stress relaxation test consisted of 4 incremental strain steps. In each step, the constructs were subjected to a 10% strain ramp at the rate of 50% strain/s and followed by 20 min relaxation under constant strain. All tested constructs reached equilibrium in the given relaxation period. Forces were recorded as a function of time, and stress was calculated by normalizing force to construct cross-section area. The peak modulus was calculated by dividing the maximum stress increment immediately after the compression increment by 10%, the strain increment.

### Statistical Analysis

GraphPad Prism 8 was used to perform statistical analysis. The paired two-sample *t*-test was used to analyze the significance level between each sample group (8020 vs. COL, 7030 vs. COL, 7030 vs. 8020) in cell viability and in gene expression after taking the replicate means within donors. Two-way ANOVA was used to determine the statistical differences in stress relaxation tests for various bioinks at different strains with Tukey’s test for multiple comparisons. The results were presented as mean ± standard deviation (SD).

## Results

### Rheology and Printing Fidelity of TCNF/ALG Precursors

**FIGURE 2:**
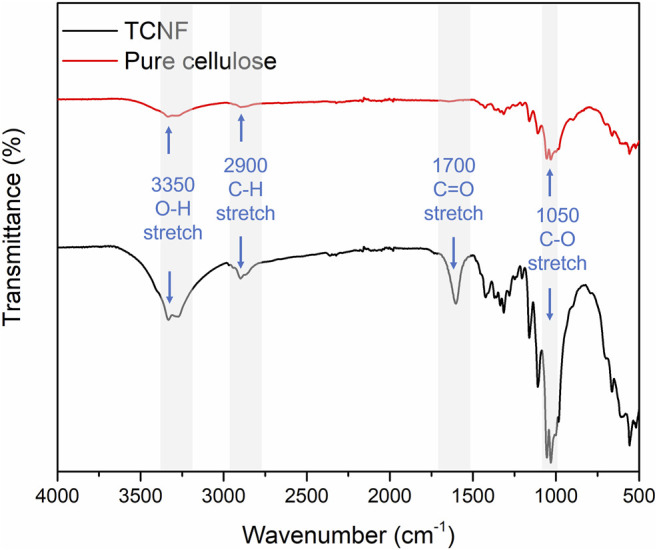
Fourier transform infrared spectroscopy of the pure cotton cellulose and TCNF.

Figure 2 presents the FTIR spectra of unmodified pure cellulose and TCNF. After TEMPO-oxidation, the characteristic absorption band of carboxyl group (C==O) stretching appeared around 1,650 cm^−1^, which is assigned to the formation of COO groups after the oxidation and release of nanofibers (El Bakkari et al., 2019). The enhancement of the -OH stretching group around 340 cm^−1^, C-H at around 2,900 cm^−1^, and C-O-C at around 1,050 cm^−1^ were also observed in TCNF, which indicated more -OH, C-O-C group are exposed.

**FIGURE 3:**
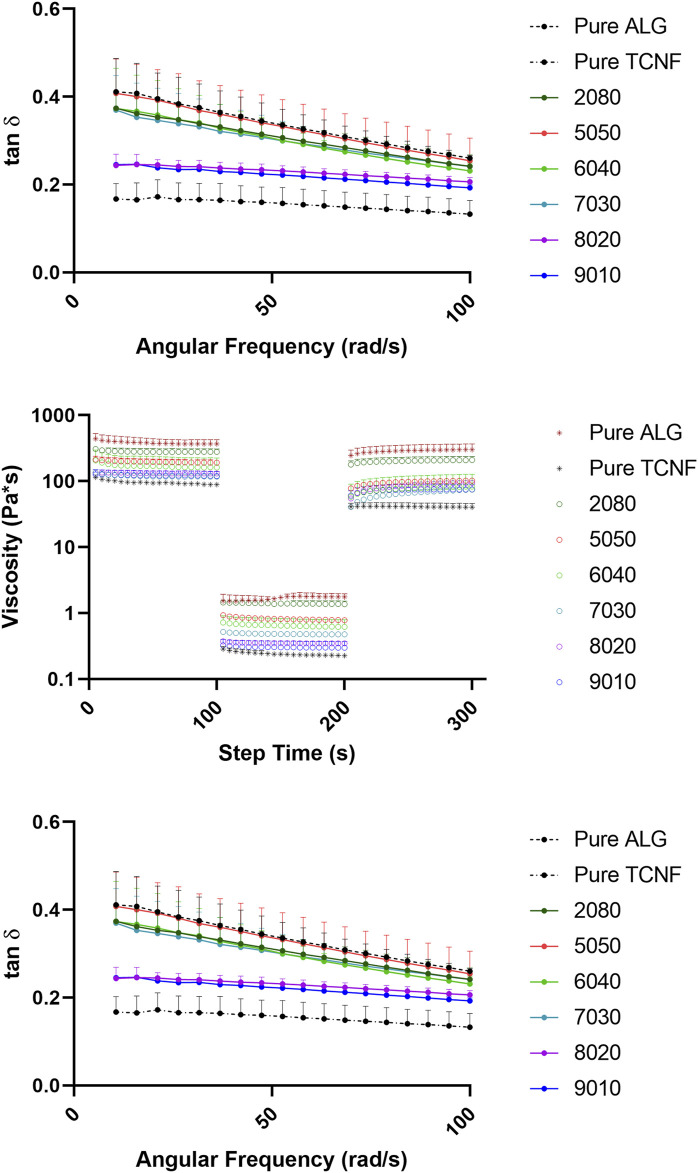
The rheology behaviors of the TCNF/ALG precursors. **(A)** The steady state flow sweeps, strain rate from 0.001 to 1,000 s^−1^. **(B)** Three-step recovery and thixotropic behavior, low shear rate at 1 s^−1^ for 100 s for initial and final step, high shear rate at 1,000 s^−1^ for 100 s for middle step. **(C)** Loss tangent of the TCNF/ALG precursors from 1 to 100 rad/s. The formulations are described in Table 1.

The rheology data of the TCNF/ALG precursors were measured to pre-estimate the printability as potential bioink materials. The viscosity vs. shear rate (steady-state flow sweep) of all the TCNF/ALG precursors is shown in Figure 3A. All TCNF/ALG precursors exhibited shear-thinning behaviour, in which the viscosity decreased as the shear rate increased. The Power Law model was also used to fit the viscosity vs shear rate curve (0.01–1,000 s^−1^) to describe the shear-shinning behaviour. The printing pressure of each TCNF/ALG precursors are shown in Table 4. The viscosity and shear rate relationship can be described in the equation of the *η* = K $\dot{\gamma }$ ^n−1^, where *η* is viscosity, $\dot{\gamma }$ is the shear rate, K and n are the empirical curves fitting parameters, known as the flow consistency index and flow behaviour index (Middleman, 1977). The fitted power-law parameters (K and n) are shown in Table 4. For shear thinning fluid, n always less than 1. The lower the n, the better the shear-thinning behaviour responding to the increase of shear rate. TCNF, 8020, 9010 exhibited and the best shear thinning behaviour, and 5050 exhibited the worst shear-thinning behaviour.

**TABLE 4:** Fitted Power-Law parameters, thixotropy recovery rate, printing pressure, filament diameter, and contractility after crosslinking.

	ALG	2080	5050	6040	7030	8020	9010	TNCF
K	432.90	503.13	232.78	194.70	237.04	137.63	155.43	156.43
n	0.24	0.23	0.25	0.19	0.18	0.13	0.14	0.16
Recovery (%)	81.6 ± 5.6	75.2 ± 2.0	53.4 ± 1.6	50.2 ± 13.0	63.7 ± 4.6	71.8 ± 4.1	62.1 ± 2.1	45.1 ± 3.6
Pressure (kPa)	200	155	125	75	75	55	55	—
Filament Diameter	—	—	0.88 ± 0.02	1.04 ± 0.08	0.79 ± 0.02	0.72 ± 0.00	0.76 ± 0.02	—
Contraction (%)	29.5%	28.8%	15.3%	10.2%	9.5%	4.3%	1.0%	—

*The filaments of the mesh printed by pure ALG and 2080 bioink material fused together and their diameter could not be measured.

The recovery and thixotropic properties were determined by applying a steady shear rate of 1 s^−1^ for 100 s suddenly increasing the shear rate to 1,000 s^−1^ for 100 s, and then reducing it to 1 s^−1^ for 100 s. Figure 3B depicts the thixotropic behaviour of the prepared hydrogels. The viscosity of the all the TCNF/ALG precursors rapidly dropped with an applied shear (printing stage) and recovered quickly after the shear force was removed (post-printing stage). However, all the samples were thixotropic and decreased in viscosity after recovery. The average viscosities of the TCNF/ALG precursors under the three shear rate stages are recorded in Supplementary Table S1. The recovery rates (initial/final viscosity) are shown in Table 4.

The frequency sweep tests were performed within the linear viscoelastic deformation region. Loss tangent (tan *δ*), the ratio of loss moduli (G″), and storage moduli (G″) for TCNF/ALG precursors as a function of frequency are shown in Figure 3C. The loss tangent (tan *δ*) determines if a material is solid-like or liquid-like. All the TCNF/ALG precursors showed a solid-like behaviour (G’>G″, tan *δ*< 1). The pure TCNF, 8020, and 9010 showed the most solid-like behaviour followed by 6040, 7030, 2080, and pure ALG and 5050 showed the least solid-like behaviour.

**FIGURE 4:**
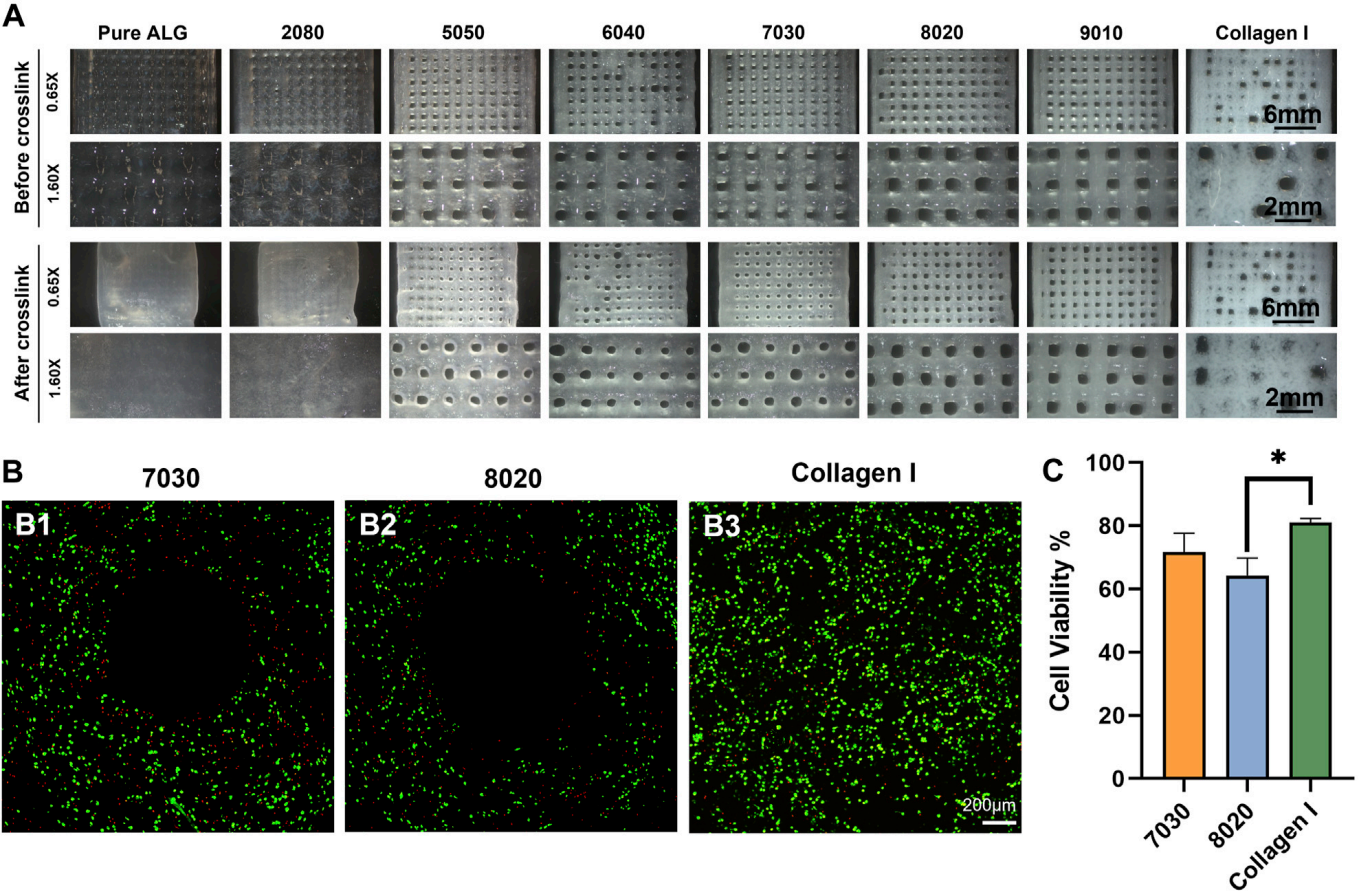
**(A)** Printed mesh structures using different formulations of TCNF/ALG precursors. **(B)** Live/dead images of the **(B1)** 7030, **(B2)** 8020, and **(B3)** COL bioinks. Live cells appear green and dead cells red. **(C)** Quantitative cell viability of the Live/Dead assay images.

The TCNF/ALG precursors were then bioprinted into mesh shapes and crosslinked using 100 mM of CaCl_2_ solution. The bioprinted constructs before and after crosslinking are shown in Figure 4A. The printing pressure, filament diameter, and contractility after crosslinking are shown in Table 4. Apparent size and shape contractions are observed in the TCNF/ALG precursors with low or no TCNF content (0100, 2080, 5050, 6040). On the other hand, the 7030, 8020, and 9010 showed the lowest contractility (≤10%) and best printing fidelity (finest filament diameter). Due to the relatively low alginate content, the 9010 was too soft to retain its shape during transfer or movement of the construct. Therefore, the 7030 and 8020 formulations were selected as bioinks to mix with hMFCs for further biochemical and biomechanical evaluations.

The LIVE/DEAD assay images after bioprinting using a 22 G needle are shown in Figure 4B. No statistically significant difference was observed between 7030 and 8020 bioinks (Figure 4C). The collagen I bioink showed a significantly higher cell viability compared to the 8020 bioink (Figure 4C).

### Histological and Biochemical Assessments

**FIGURE 5:**
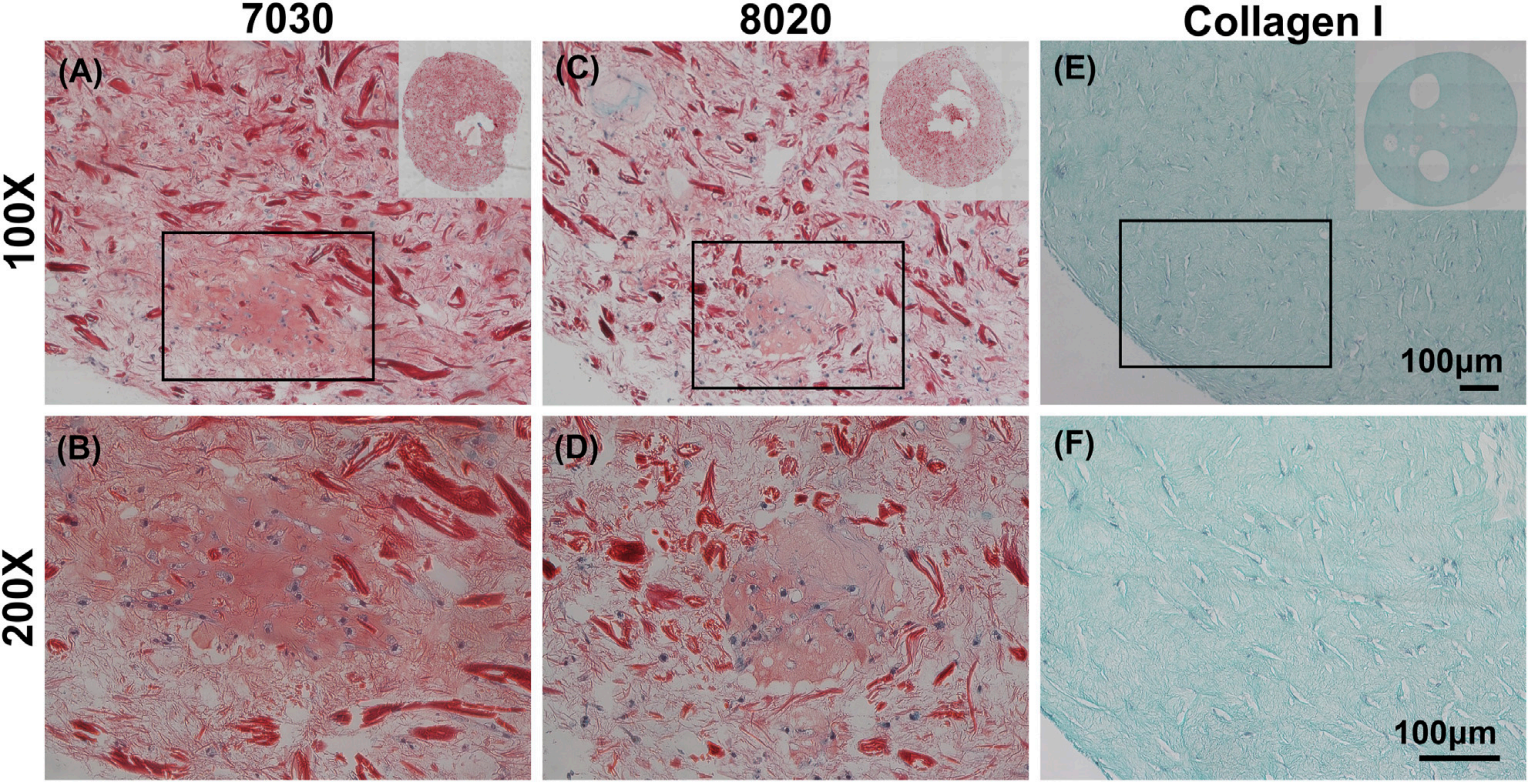
Safranin-O staining (red, stained negatively charged proteoglycans and the TCNF) with Fast Green counterstain (green, stained proteins) and cells stained with hematoxylin of **(A,B)** 7030, **(C,D)** 8020, and **(E,F)** COL I bioinks. Scale bar is 100 μm.

It is commonly understood that Safranin-O would only stain when sulfated GAG is present within the sample. Interestingly, we discovered that cellulose nanofibers (TCNF) could also be stained by Safranin-O despite lacking sulfated GAG, as shown in Supplementary Figure S1 in supplemental materials. Previous studies which used CNFs as biomaterials for cartilage tissue engineering did not report and discuss this phenomenon (Nguyen et al., 2017). Through comparing against cell-free CNF samples, it was still possible to distinguish the Safranin-O positive matrix throughout the histological sections of two TCNF/ALG bioink (Figures 3A,B). In contrast, the bioprinted COL bioink did not show any positive safranin-O positive matrix deposition throughout the section (Figures 5E,F).

**FIGURE 6:**
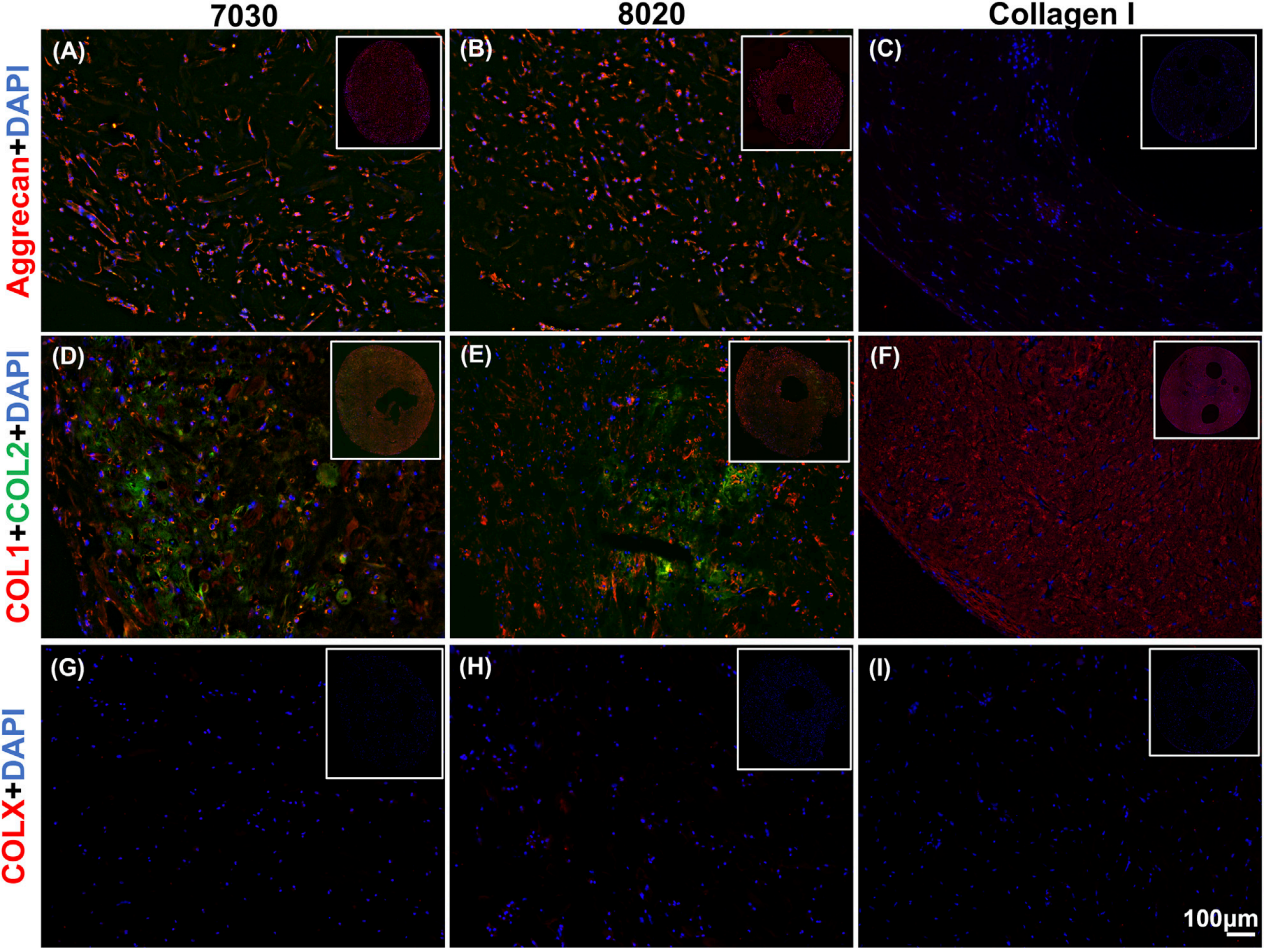
Immunofluorescent staining of **(A–C)** of aggrecan (red), **(D–F)** collagens I and II (red and green, respectively), and **(G–I)** collagen X. The staining of nuclei is blue. Scale bar is 100 μm.

**FIGURE 7:**
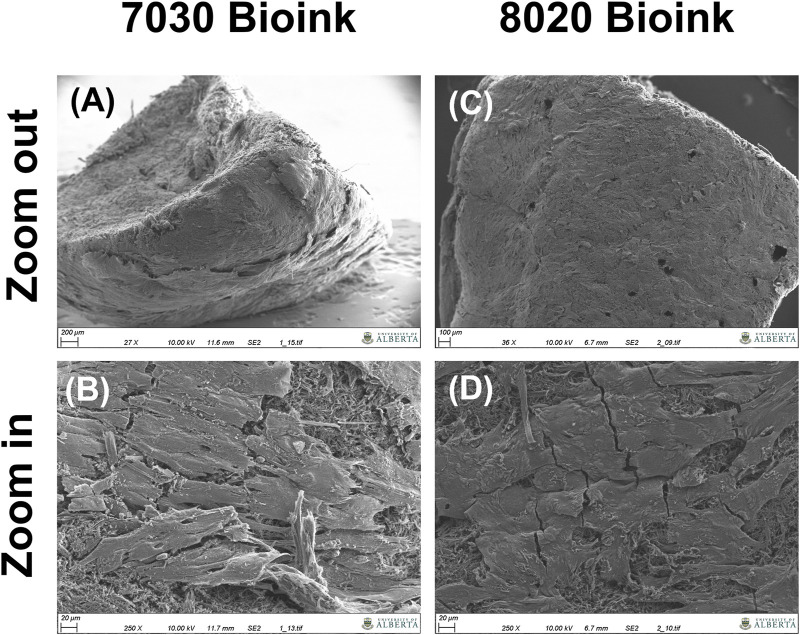
SEM images of the bioprinted constructs after 6 weeks of *in vitro* culture, **(A,B)** the cross-section of the 7030 bioink **(C,D)** surface of the 7030 bioink.

Positive aggrecan immunofluorescent staining was shown in both TCNF/ALG bioinks (Figures 6A,B) but not in the COL bioink (Figure 6C), which was consistent with the Safranin-O staining results. The immunofluorescence staining also showed a universal presence of collagen I in two TCNF bioinks (Figures 6D,E) and the COL bioink (Figure 6F). The COL bioink showed more densely packed collagen I compared to the two TCNF/ALG bioinks. However, collagen II was present in the two TCNF/ALG bioinks (Figures 6D,E) and not in the COL bioink (Figure 6F). The collagen II matrix was most concentrated at the outer edges of the constructs. There was no detectable collagen X immunofluorescence in any constructs (Figures 6G–I). The SEM images of the 7030 and 8020 bioinks after 6 weeks of *in vitro* chondrogenic culture are depicted in Figure 7. Newly synthesized ECM was observed on the surface of the bioprinted constructs for both 7030 and 8020 bioinks. The TCNF material was visible under the ECM.

**FIGURE 8:**
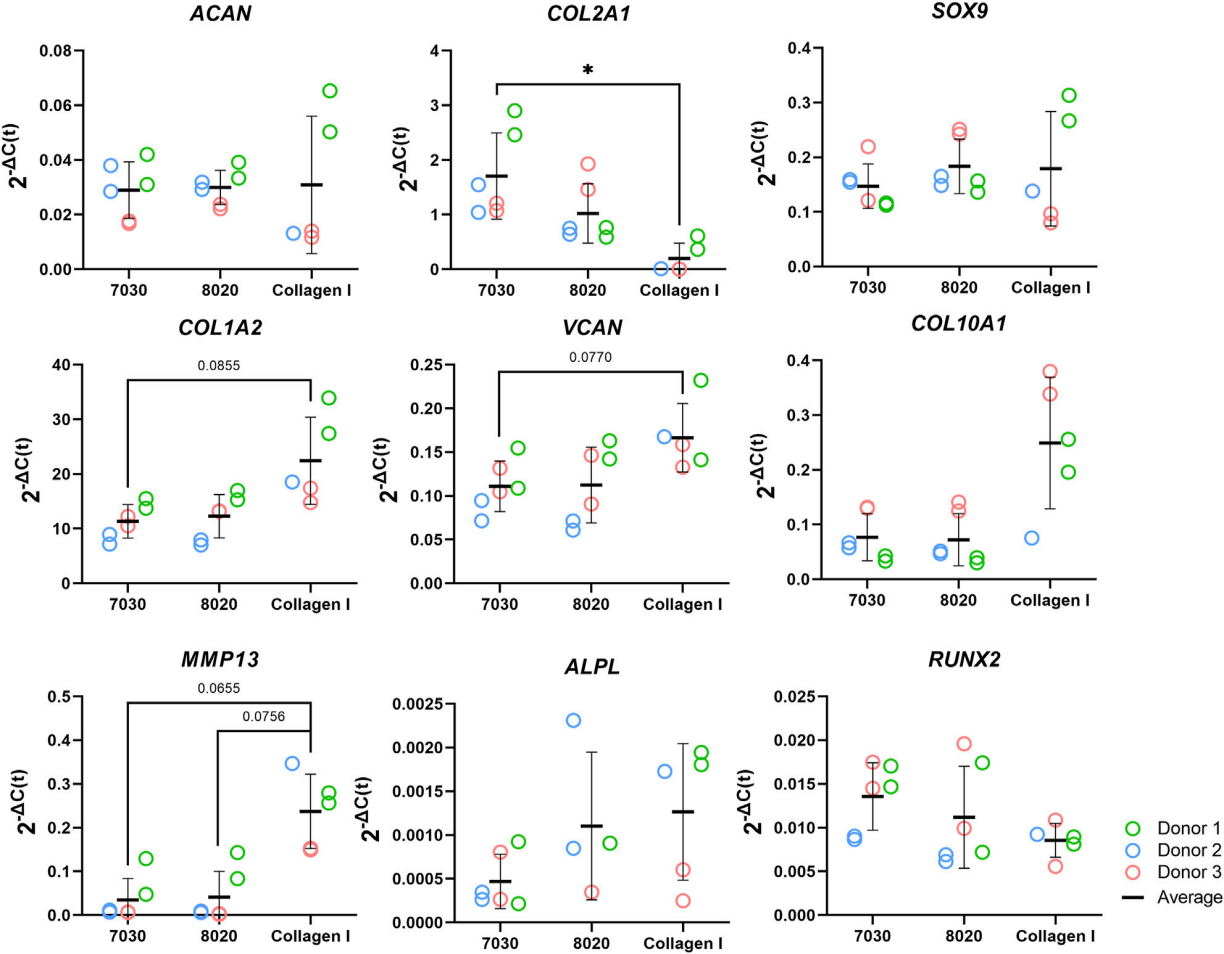
Fibrocartilage related gene expression of bioprinted constructs after 6 weeks of *in vitro* chondrogenic culture (*n* = 3). * represents *p* < 0.05.

The mRNA expression of fibrocartilage-related (*ACAN*, *COL1A2*, *COL2A1*, *SOX9,* and *VCAN*) and bone-related (*COL10A1, MMP13, ALPL,* and *RUNX2*) genes are shown in Figure 8. For all these genes, no significant differences were observed between the 7030 and 8020 bioinks. The fibrous markers *COL1A2* and *VCAN* in the COL bioink were higher than in 7030 bioinks with near statistical significance (i.e., *p* = 0.086 and *p* = 0.077, respectively). The cartilaginous marker *COL2A1* in the COL bioink was significantly lower compared to the 7030 bioink. No statistically significant difference was observed among the three bioinks in gene expression of *ACAN* and *SOX9*. No significant difference was noted in *ALPL*, and *RUNX2* among the three groups for these bone formation-related genes. On the other hand, there was an upregulation of *MMP13* in the COL bioink compared to 7030 and 8020 with near statistical significance (i.e., *p* = 0.066 and *p* = 0.076, respectively). The expression of *COL10A1* in the COL bioink for each donor increased but with no detectable significant difference.

**FIGURE 9:**
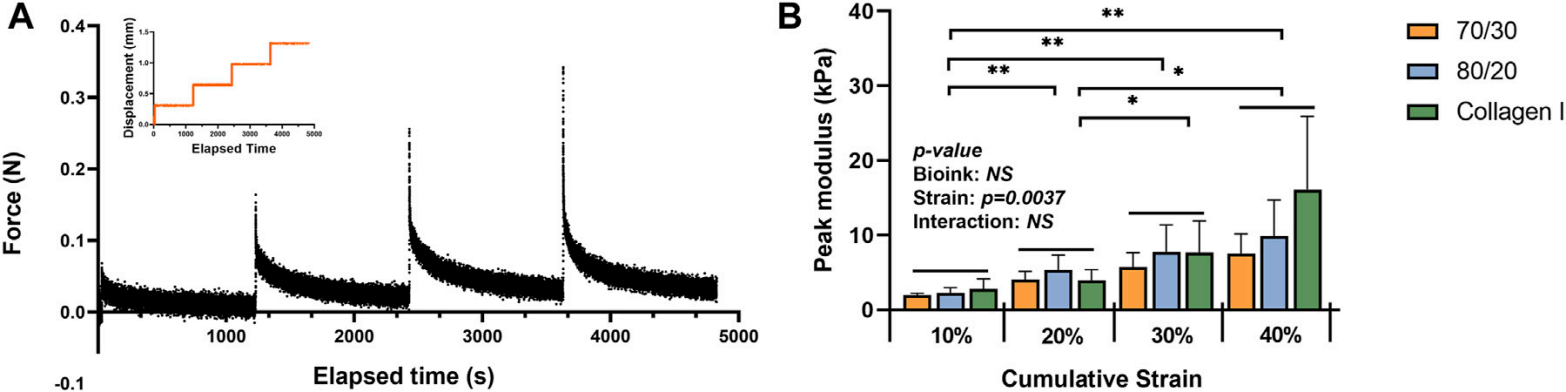
Mechanical properties of the bioprinted constructs: **(A)** an example of data acquisition of the strain-controlled unconfined compression test **(B)** the peak modulus as a function of the cumulative strain (*n* = 3). * represents *p* < 0.05.

After the strain-controlled unconfined compression test, the peak modulus (instantaneous modulus) was calculated using the force change between the peak and equilibrium forces at each relaxation period. The calculated values were normalized by the cross-sectional area of the 3D bio-printed tissues. The stepwise stress-relaxation as a function of time of one donor (0–10%, 10–20%, 20–30%, and 30–40% strain for each force jump) and the peak modulus as a function of the cumulative strain is shown in Figures 9A,B. No significant interaction was observed between material type and strain in the two-way ANOVA. Therefore, only the main effects of each factor, i.e., strain and bioink material, were tested. Strain was the only significant main effect for the compressive modulus, with the significant differences between each strain level, except for 30% vs. 40%.

## Discussion

The newly appeared carboxyl group (C=O) indicated the successful oxidation of the cellulose using TEMPO. The exposure of O-H and C-O-C groups may be due to the mechanical processing of the cellulose. The smaller size of the cellulose fibres allowed for exposure of more surface groups.

In the steady-state flow sweep test (Figure 3A), all TCNF/ALG precursors exhibited a shear-thinning behaviour, which may be attributed to the loss of chain entanglement as well as the alignment of polymeric chains/fibre under steady-state shear. As the shear rate increases, the entanglement of the hydrogel networks is weakened and the entrapped liquid that resists the flow is released, and therefore induce decreased viscosity. Better shear-thinning behaviour is desired for the 3D bioprinting process, since under fixed printing speed and needle size lower bioink viscosities in the printing needle will result in lower shear stress experienced by cells, resulting in better cell viability. The formulation of the TCNF/ALG precursors has a strong effect on the shear-thinning behaviour. For alginate rich TCNF/ALG precursors (TCNF content between 0 and 50%), increasing the TCNF content did not show a significant change in the shear thinning behavior of the fluid. In contrast, for TCNF-rich TCNF/ALG precursors (TCNF content between 50 and 100%), a higher TCNF content led to better shear-thinning behaviour. Under fixed printing speed, the required printing pressure decreased dramatically as the TCNF content increased due to the enhanced shear-thinning effect (Table 4).

The thixotropic property is an important parameter to predict printing fidelity. Abouzeid et al. (2018) found that the higher the recovery rate (initial/finial viscosity) of the bioinks, the better the printing fidelities. They found the printed scaffolds had the best printing fidelity with the most uniform widths and stable shapes at 66% recovery rate, which is the highest recovery rate among the investigated bioink formulations (Abouzeid et al., 2018). However, our results only partially agreed with their findings. The pure ALG (81.6%), 2080 (75.2%), 8020 (71.8%), 7030 (63.7%), and 9010 (62.1%) showed a relatively high recovery rate (recovery >60%). Only 7030, 8020, and 9010 exhibited uniform width and clear edges throughout the printed shape. The filaments printed by pure alginate precursors, which has the highest recovery rate, collapsed and fused with neighbouring filaments. These results show that some other rheology parameters can affect printability as well. Gao et al. (2018) stated that the loss tangent is an important parameter for predicting structural integrity and extrusion uniformity. For pure alginate precursors, the tan *δ* is 0.41 in the low-frequency range (10 rad/s), which is in the relatively high loss tangent range stated by Gao et al. (2018). The high loss tangent represents lower structural integrity (the collapse and fusion of the bioprinted structure), but better extrusion uniformity. The high tan *δ* explains the reason why pure alginate exhibits poor printing fidelity even it has a high thixotropic recovery rate. This statement is further validated in the bioprinting of collagen I gel. In our previous study, the collagen I gel showed more liquid-like behaviour, requiring it to be printed in a support bath (Lan et al., 2021). In this study, we directly printed the collagen I gel without the supporting bath to compare the printability with TCNF/ALG precursors. We found that the printed collagen I filaments tend to spread out after bioprinting, which resulted in poor printing fidelity.

It is also important for the bioink to retain its original shape during the crosslinking stage. We found that the alginate-rich TCNF/ALG precursors (0100, 2080, 5050, 6040) all exhibited high contractility after printing (≥10%). The contraction of the bioprinted constructs may contribute to the stability of the swelled alginate polymer chains in water after crosslinking. The alginate polymers are first dispersed and swelled in water. By adding the Ca^2+^ ions to alginate, the Ca^2+^ cations are coordinately bound to the COO^−^ group in the alginate polymer chain and arranged into an “egg-box” model (Braccini and Pérez, 2001; Li et al., 2007a). The 7030, 8020, and 9010 showed the best printing fidelity and lowest contractility after bioprinting. Due to the low alginate content of 9010, changing in shape is observed during transporting the bioprinted construct using 9010 (the distortion of the printed shape); therefore, only 7020 and 8020 were mixed with hMFCs (7030, 8020 bioinks) to generate tissue-engineered fibrocartilages.

A LIVE/DEAD assay was performed to investigate the biocompatibility of the bioprinted constructs. The COL bioink showed higher cell viability and higher cell numbers compared to the TCNF/ALG bioinks. It is speculated that the higher cell viability with the COL bioink is due to the relatively lower printing pressure, the presence of cell adhesive motifs in the protein-based hydrogel, and that the hMFCs’ natural host material is collagen I.

After 6 weeks of culture, hMFC-deposited ECM was observed in both 7030 and 8020 bioinks under SEM (Figure 7). A dense layer of ECM covered the cellulose nanofibers. The histology and immunofluorescence results further confirmed the ECM formation in the 7030 and 8020 bioinks. The staining results suggest that the hMFC-laden TCNF/ALG bioinks (7030, 8020) support development of a more inner meniscus fibrocartilage-like phenotype (presence of sGAG, collagen I and II), while the (bovine) COL bioink supports a more outer meniscus phenotype of no Safranin-O positive ECM with the presence of newly-synthesized human collagen I. Neither the cellulose nanofibers nor the alginate present a natural cell binding site. The lack of a cell-binding site forces the monolayer-expanded hMFCs into a round shape known to promote a chondrogenic phenotype (Daly et al., 2016). In contrast, collagen I presents natural cell-binding motifs through its interaction with the integrins that provide focal adhesion points to the hMFCs (Wang et al., 2020a). The focal adhesions can support cell spreading, which may facilitate the development of more outer meniscus-like phenotypes. The fibrocartilage matrix phenotypes at the mRNA level also agree with the histological and immunofluorescence findings (Figure 8).

Our observations agree with Daly et al. (2016)’s finding that alginate and agarose bioinks (bioinks without cell-binding sites) support the development of hyaline-like cartilage tissue, while GelMA and PEGDA bioinks (bioinks with a native binding site) better support the development of fibrocartilage-like tissue. As shown in Figure 8, the lack of cell-binding sites in the TCNF/ALG bioinks correlated with reduced expression of *COL10A1* and *MMP13* (indicators of hypertrophic chondrocytes during endochondral ossification). The upregulation of these two genes is more likely to occur when integrin-binding (α2 integrin) between hMFCs and collagen I is present. The α2 integrin is a subunit of the most known collagen I receptor (α2β1). The interaction between this α2 integrin subunit and collagen I facilitates osteoblastic differentiation, an important event in the expression of the osteogenic phenotype (Mizuno et al., 2000; Bosnakovski et al., 2006).

Although proteoglycans mainly contribute to a tissue’s compressive strength, the bioprinted TCNF/ALG bioink constructs did not show a higher compressive modulus compared to the COL bioink-based constructs. The average compressive modulus at the 30–40% strain step was higher than in the two TCNF/ALG bioinks, but no significant difference was observed. Hence, the engineered matrix composition does not fully correspond to the mechanical properties of the engineered tissue. One plausible explanation is that the crosslinking of the COL bioink constructs was augmented via endogenous lysyl oxidase expression, which can be induced by hypoxic culture (Makris et al., 2013). The cations that crosslink alginate chains may also be released into the culture media and washed away during the media changes, which may diminish the mechanical properties of the TCNF/ALG constructs.

One limitation of the present study is the low number of human meniscus donors used, leaving uncertainty regarding donor-to-donor differences. Another limitation is the lack of an *in vivo* study of the bioprinted constructs. The *in vivo* stability of the deposited ECM within the constructs in regard to *in vivo* calcification, bone formation, vascularization, and the retention of the Safranin-O positive ECM after implantation merits future investigation.

## Data Availability

The original contributions presented in the study are included in the article/Supplementary Materials, and further inquiries can be directed to the corresponding author.
