# Supplementary material for: TEMPO-Oxidized Cellulose Nanofiber-Alginate Hydrogel as a Bioink for Human Meniscus Tissue Engineering
Source: Front Bioeng Biotechnol. 2021 Nov 5;9:766399. doi: 10.3389/fbioe.2021.766399 (PMC8602093; doi:10.3389/fbioe.2021.766399)
Supplement: Supplementary file 1 [file DataSheet1.docx]

**Supplementary Figure 1**.

Safranin-O staining of the empty TCNF/ALG scaffold (A) 7030 (B) 8020. Scale bar is 100 μm


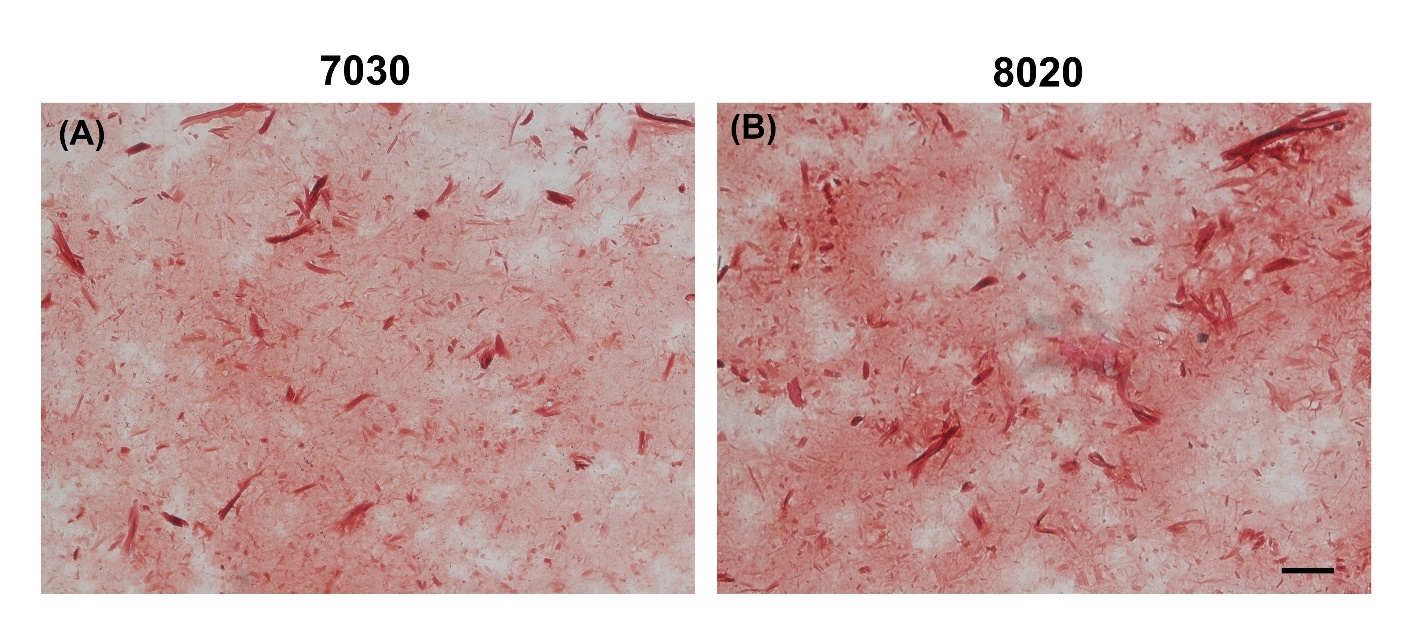


**Supplementary Table 1**.

Thixotropic viscosities of TCNF/ALG bioinks. The η is the measured viscosity for each shear step (1 s^-1^, 1000 s^-1^, 1 s^-1^) at 100 s.

|  | Pure ALG  (0100) | 2080 | 5050 | 6040 | 7030 | 8020 | 9010 | Pure TCNF |
| --- | --- | --- | --- | --- | --- | --- | --- | --- |
| Initial low strain phase η (Pa*s)  Shear rate $\dot{\gamma}$ = 1 s^-1^ | 367.0±47.3 | 277.6±25.5 | 191.3±15.7 | 161.8±52.0 | 117.2±2.5 | 131.6±6.3 | 120.0±17.0 | 88.9±5.3 |
| High strain phase η (Pa*s)  Shear rate $\dot{\gamma}$ = 1000 s-1 | 1.8±0.2 | 1.4±0.1 | 0.8±0.0 | 0.6±0.1 | 0.5±0.0 | 0.30±0.0 | 0.3±0.1 | 0.2±0.0 |
| Finial low strain phase η (Pa*s)  Shear rate $\dot{\gamma}$ = 1 s-1 | 301.5±54.0 | 209.3±23.4 | 102.0±6.0 | 82.9±36.4 | 74.5±2.8 | 94.5±5.6 | 74.9±12.4 | 40.2±4.6 |
